# Analyzing Spatial and Temporal Patterns of Designated Malaria Risk Areas in Nepal from 2018 to 2021

**DOI:** 10.1089/vbz.2022.0097

**Published:** 2023-06-05

**Authors:** Shreejana Bhattarai, Jason K. Blackburn, Sadie J. Ryan

**Affiliations:** ^1^ Quantitative Disease Ecology and Conservation (QDEC) Lab, Department of Geography, University of Florida, Gainesville, Florida, USA.; ^2^ Emerging Pathogens Institute, University of Florida, Gainesville, Florida, USA.; ^3^ Spatial Epidemiology and Ecology Research (SEER) Laboratory, Department of Geography, University of Florida, Gainesville, Florida, USA.

**Keywords:** Nepal, risk stratification, malaria, spatial and temporal trend

## Abstract

**Background:**

Nepal is preparing to eliminate malaria by 2026. To evaluate the progress of vector control and prioritize areas for targeted intervention, understanding the recent changing distribution of high and moderate malaria risk areas is vital.

**Methods:**

Patterns of designated high and moderate malaria risk wards in Nepal between 2018 and 2021 were analyzed to identify stable and newly generated high- and moderate-risk (HaMR) wards, using the Spatial Temporal Analysis of Moving Polygons (STAMP) method.

**Results and Conclusions:**

High-risk and moderate-risk wards decreased by about 55% and the number of districts containing these wards also decreased from 20 to 14. However, several stable and new HaMR wards, mostly in the northwest and the southwest of the country, are apparent, despite intervention efforts. Public health officials should prioritize those wards for malaria surveillance and vector control, and future studies should explore the underlying reasons for persistent risk wards.

## Introduction

Malaria was a major public health issue in Nepal for much of the 20th century (Newby, 2015), but cases declined significantly in the past two decades, in response to concerted intervention efforts, as part of a push to elimination. Between 2010 and 2020, indigenous malaria declined by about 98% with only 73 cases reported in Nepal in 2020 (EDCD, 2021), and Nepal is preparing for malaria elimination by 2026. As malaria incidence decreases in response to intervention coverage, cases and transmission rates become heterogenous across the country.

In this phase, a key approach to optimize malaria intervention is “stratification” in which the country or region is divided into smaller units based on the malaria risk (WHO, 2015). Malaria risk stratification identifies geographical areas at potential risk of malaria transmission to guide targeted intervention and for an effective and efficient resource mobilization (EDCD, 2018a; EDCD, 2018b). Nepal conducted its first malaria risk stratification in 2010 at the district level based on annual parasite index (API), which is the total number of confirmed malaria cases per 1000 population at risk of malaria (DoHS, 2010; DoHS, 2009).

This stratification defined four risk categories: high-risk (HR), moderate-risk (MR), low-risk, and no-risk. With the large decline in burden, and evidence of malaria-endemic and malaria-free areas occurring within the same district, malaria stratification was refined to the subdistrict level (subdistrict geographic units called village development committees [VDCs]), in 2013. This stratification was based on disease burden, ecology, and vulnerability (Rijal et al., 2019). The continued declines showed that within a VDC, malaria activity is concentrated in some wards (subdivisions of VDCs), whereas others remain free. Risk stratification was refined to wards in 2016, and has been reported annually since 2018, using the same categories.

In this study, we analyzed the interannual spatiotemporal patterns of high- and moderate-risk (HaMR) wards from 2018 to 2021 by using the Spatial Temporal Analysis of Moving Polygons (STAMP) method. Identifying areas of stable or newly generated high- or moderate-risk areas identifies persistence of malaria or emergence of new risk areas. The objective was to identify priority areas for malaria surveillance and vector control as Nepal prepares for malaria elimination by 2026.

## Materials and Methods

### Malaria risk areas data

The names of HaMR malaria wards for the years 2018–2021 were obtained from reports published online by the Epidemiology and Disease Control Division (EDCD) of the Department of Health, Government of Nepal. Using a shapefile of the wards in Nepal (HES, 2020), we assigned the two categories of malaria risk to the wards: HR and MR wards and created a shapefile in ArcGIS 10.6.1 (ESRI, Redland, CA).

### Spatial temporal analysis of moving polygons

To quantify patterns and changes of HaMR wards in Nepal, we used the STAMP algorithm (Robertson et al., 2007). STAMP overlays polygons from two time periods and evaluates changes in spatial position (Bezymennyi et al., 2014; Morris et al., 2013). The analysis includes the identification of regions newly generated (emerging risk), remaining stable (persistent risk) or disappeared (reduced risk). Polygons remaining at the same risk in two consecutive time periods are classified as “stable.” Polygons at risk in the first time but absent in the second are classified as “disappearance.”

Polygons at risk only in the second time are classified as “generation” (here “new”). Separate STAMP analyses were performed for each risk category: HR and MR for each interannual period: 2018–2019, 2019–2020, and 2020–2021. Risk category changes within wards were also identified, such as moderate to high or high to moderate between years, using STAMP across categories (Supplementary Data). STAMP analysis was performed in R version 4.1.3 (2022-03-10) using the “stampr” package. All final maps were produced in ArcGIS.

## Results

**FIG. 1.**
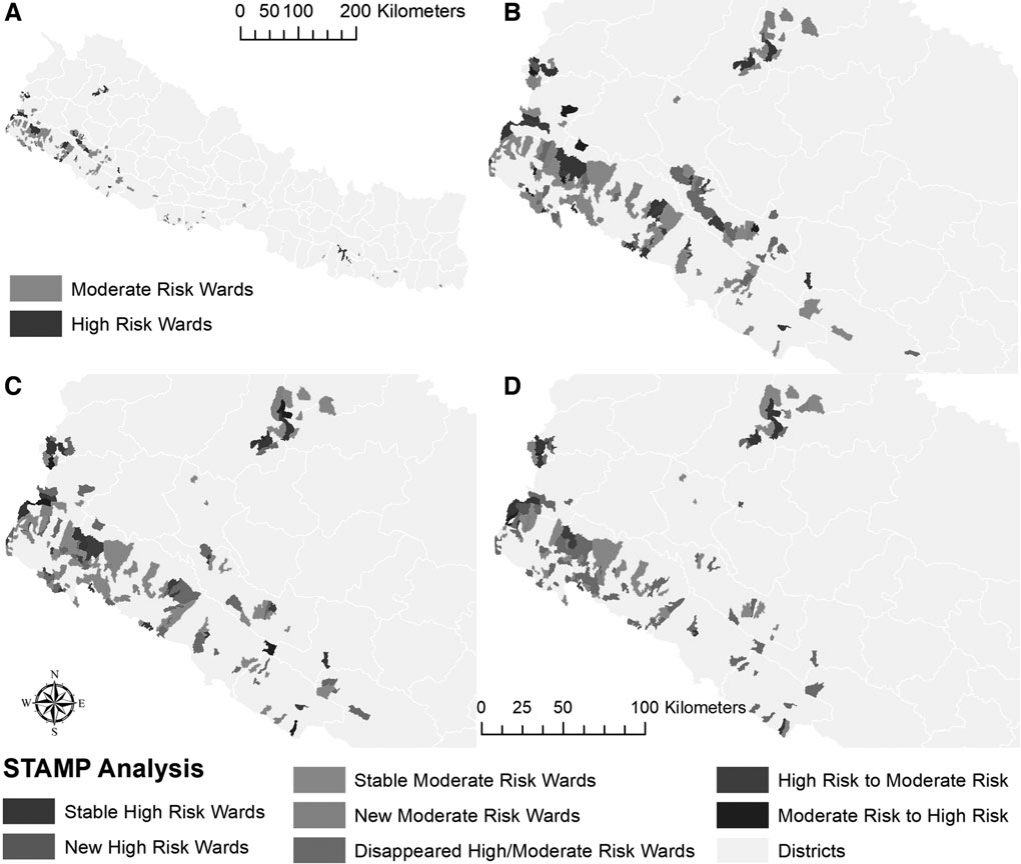
STAMP analysis for high- and moderate-risk malaria wards in Nepal between 2018 and 2021. **(A)** Distribution of high- and moderate-risk wards in 2018. **(B)** Results of STAMP analysis between 2018 and 2019 in western Nepal. **(C)** Results of STAMP analysis between 2019 and 2020 in western Nepal. **(D)** Results of STAMP analysis between 2020 and 2021 in western Nepal. The STAMP analysis for the whole country is presented in Supplementary Fig. S1. STAMP, Spatial Temporal Analysis of Moving Polygons.

From 2018 to 2021, the number of HaMR wards decreased by about 55% in Nepal (Supplementary Table S1). There were 153 MR and 49 HR wards in 2018, which decreased to 68 MR and 22 HR wards in 2021. The HaMR wards were primarily concentrated in the southwest of Nepal in 2018 with very few HaMR wards in the northwest and the rest of the country (Fig. 1A). Between 2018 and 2019, several new MR wards appeared in the northwest (Fig. 1B) and remained stable. In the southwest region, several HaMR wards disappeared, whereas others persisted (Fig. 1B, D). MR wards were more widespread and persistent than HR wards in Nepal, and some wards changed from MR to HR and vice versa (Fig. 1B–D). The number of wards changing category decreased across the years of the study (Supplementary Table S4).

There were 20 districts with HaMR wards in 2018. Humla and Achham districts had new HaMR wards in 2019, whereas Jhapa and Sarlahi became free of HaMR wards in the same year. In 2020, Bara, Dhanusha, Rupandehi, and Sindhuli became free of HaMR wards. In 2021, Dang, Nawalpur, and Salyan became free of HaMR wards, whereas Kalikot had new HR wards. This resulted in 2021 having 14 districts with HaMR wards. Humla and Achham had stable HaMR wards in 2021, which had been free of HaMR wards until 2018. Six HR wards in three districts and 20 MR wards in four districts remained consistently stable throughout the study period (Supplementary Table S5). The number of stable and new HaMR wards gradually decreased over the years, and disappeared HaMR wards increased (Supplementary Tables S2 and S3).

## Discussion

Interannual patterns of malaria risk in Nepal between 2018 and 2021 were analyzed using the STAMP algorithm at the ward level. The implementation of vector control interventions has decreased the number of HaMR wards in Nepal, and the number of districts with HaMR wards has also decreased. This result is in agreement with studies done in other countries. For example, after vector control interventions, the number of high transmission districts dropped from 14.3% to 6.4% in Ethiopia between 2013 and 2016 (Taffese et al., 2018).

Similarly, in Bhutan, the geographical distribution of malaria infection decreased from 15 of the 20 districts in 2006 to only 2 districts in 2018 (Wangchuk et al., 2019). Although the number of HaMR wards decreased in Nepal, there were several stable HaMR wards throughout the study period, meaning malaria is still transmitted in these areas, despite vector control. Future studies should examine conditions associated with stability of these HaMR wards. In addition, we detected several new HaMR wards, mostly in the northwest and southwest.

In a previous study, we identified clusters of a significantly increasing trend of imported malaria in the southwest (Bhattarai et al., 2023), and the stable and new HaMR wards in the southwest may be due to imported malaria. Several other studies also have identified the high number of imported malaria cases in the western part of Nepal (Dhimal et al., 2014a; EDCD, 2018a; Rijal et al., 2018; Smith et al., 2019). Concurrently, several HaMR wards in the southwest disappeared during the study period, corroborating our previous study showing declining trends of several malaria indicators except imported malaria (Bhattarai et al., 2023). Some of this region may observe reduced transmission in the future with temperatures increasing to levels too high to be suitable for transmission (Bhattarai et al., 2022).

The generation of new HaMR wards in the northwest in 2019, and their stability throughout the study years is very concerning given this area falls in the hilly and mountainous region in Nepal, historically not endemic for malaria. This suggests malaria is becoming established in the mountainous area, which may be due to climate change. In a previous study, we found a significantly increasing trend of indigenous malaria in this same region (Bhattarai et al., 2023).

This area is projected to observe additional emergence and increase in season length of malaria transmission suitability in the future (Bhattarai et al., 2022), pointing to the need to prioritize these areas for surveillance, and vigilance in emerging new risk areas. Malaria transmission has increased in the highland areas in different parts of the world due to climate change (Loevinsohn, 1994; Siraj et al., 2014). In Nepal too, some studies have found that climate change has already affected malaria transmission (Badu, 2013; Bhandari et al., 2013; Dhimal et al., 2014b; Ghimire, 2016).

Few HR wards converted to MR; however, several MR wards converted to HR despite vector control intervention. Further study to understand where intervention succeeds and fails in an era of elimination, is needed.

## Conclusions

High and moderate malaria risk areas in Nepal decreased significantly in Nepal between 2018 and 2021. However, there were several stable HaMR wards and some new HaMR wards, particularly in the northwest and southwest regions. Public health officials should focus on these areas to prioritize surveillance and vector control interventions. Future studies should explore the underlying reasons for persistent risk areas in these regions.

## Supplementary Material

Supplemental data

Supplemental data

Supplemental data

Supplemental data

Supplemental data

Supplemental data

Supplemental data

## Supplementary Material

Supplementary Method
