# Supplementary material for: Analyzing Spatial and Temporal Patterns of Designated Malaria Risk Areas in Nepal from 2018 to 2021
Source: Vector Borne Zoonotic Dis. 2023 Jun 5;23(6):350–3. doi: 10.1089/vbz.2022.0097 (PMC10278016; doi:10.1089/vbz.2022.0097)
Supplement: Supplemental data [file Supp_DataS1.docx]

## Supplemental Materials

1. **Methods:**

Separate STAMP analyses were performed for each risk category: moderate-risk (MR) and high-risk (HR) and for each inter-annual period: 2018-2019, 2019-2020, and 2020-2021.

2018MR-2019MR

2018HR-2019HR

2019MR-2020MR

2019HR-2020HR

2020MR-2021MR

2020HR-2021HR

Risk category changes within wards were also identified, such as moderate to high or high to moderate between years, using STAMP across categories.

2018MR-2019HR

2018HR-2019MR

2019MR-2020HR

2019HR-2020MR

2020MR-2021HR

2020HR-2021MR

1. **References**

Newby G. Eliminating Malaria in Nepal. Global Health Group, UCSF; 2015.

Rijal KR, Adhikari B, Adhikari N, et al. Micro-Stratification of Malaria Risk in Nepal: Implications for Malaria Control and Elimination. Trop Med Health 2019;47(1):21; doi: 10.1186/s41182-019-0148-7.

Robertson C, Nelson TA, Boots B, et al. STAMP: Spatial–Temporal Analysis of Moving Polygons. J Geogr Syst 2007;9(3):207–227; doi: 10.1007/s10109-007-0044-2.

Taffese HS, Hemming-Schroeder E, Koepfli C, et al. Malaria Epidemiology and Interventions in Ethiopia from 2001 to 2016. Infect Dis Poverty 2018;07(06):1–9; doi: 10.1186/s40249-018-0487-3.

WHO. Global Technical Strategy for Malaria 2016–2030. World Health Organization; 2015.
