# Supplementary material for: Analyzing Spatial and Temporal Patterns of Designated Malaria Risk Areas in Nepal from 2018 to 2021
Source: Vector Borne Zoonotic Dis. 2023 Jun 5;23(6):350–3. doi: 10.1089/vbz.2022.0097 (PMC10278016; doi:10.1089/vbz.2022.0097)
Supplement: Supplemental data [file Supp_TableS1.docx]

**Table S1: Number of Moderate- and High- Risk wards in Nepal between 2018 and 2021**

| Year | 2018 | 2019 | 2020 | 2021 |
| --- | --- | --- | --- | --- |
| Moderate-Risk wards | 153 | 151 | 124 | 68 |
| High-Risk wards | 49 | 47 | 28 | 22 |
| Total | 202 | 198 | 152 | 80 |
