# Supplementary material for: Analyzing Spatial and Temporal Patterns of Designated Malaria Risk Areas in Nepal from 2018 to 2021
Source: Vector Borne Zoonotic Dis. 2023 Jun 5;23(6):350–3. doi: 10.1089/vbz.2022.0097 (PMC10278016; doi:10.1089/vbz.2022.0097)
Supplement: Supplemental data [file Supp_FigS1.docx]

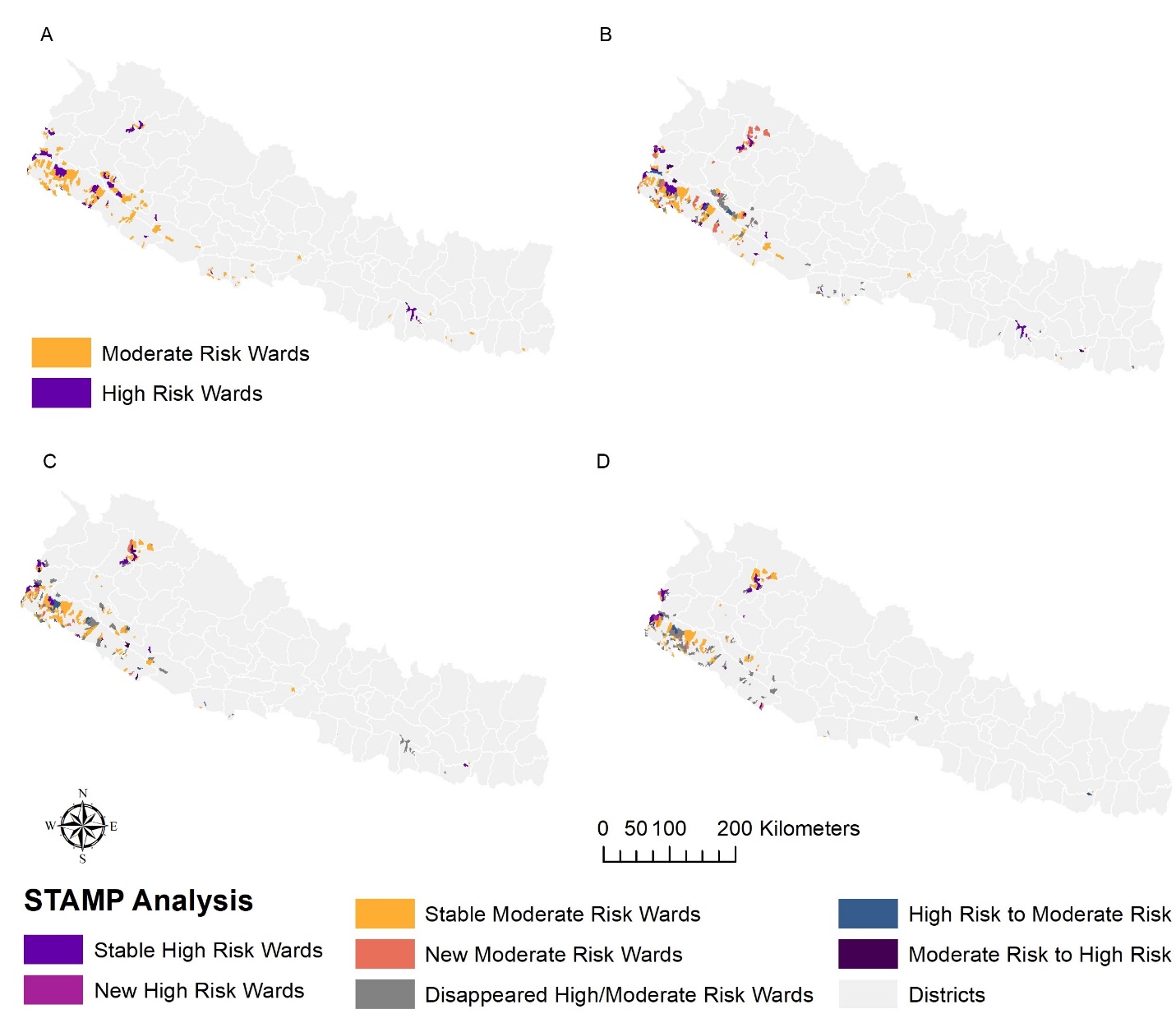


Figure S1: STAMP analysis for High- and Moderate-Risk malaria wards in Nepal between 2018-2021. A. Distribution of High- and Moderate-Risk wards in 2018. B. Results of the STAMP analysis between 2018-2019. D. Results of the STAMP analysis between 2019-2020. D. Results of STAMP analysis between 2020-2021.
