# Supplementary material for: Analyzing Spatial and Temporal Patterns of Designated Malaria Risk Areas in Nepal from 2018 to 2021
Source: Vector Borne Zoonotic Dis. 2023 Jun 5;23(6):350–3. doi: 10.1089/vbz.2022.0097 (PMC10278016; doi:10.1089/vbz.2022.0097)
Supplement: Supplemental data [file Supp_TableS5.docx]

**Table S5: STAMP-based stable High- and Moderate-Risk wards from 2018 to 2021.**

| Stable | Ward No. | GP/NP | District | Province |
| --- | --- | --- | --- | --- |
| High-Risk | 8 | Khatyad GP | Mugu | Karnali |
|  | 10 | Khatyad GP | Mugu | Karnali |
|  | 1 | Budhinanda NP | Bajura | Sudur Pashchim |
|  | 5 | Budhinanda NP | Bajura | Sudur Pashchim |
|  | 6 | Budhinanda NP | Bajura | Sudur Pashchim |
|  | 9 | Bhimdatta NP | Kanchanpur | Sudur Pashchim |
| Moderate-Risk | 6 | Himali GP | Bajura | Sudur Pashchim |
|  | 13 | Bhimdatta NP | Kanchanpur | Sudur Pashchim |
|  | 4 | Bedkot NP | Kanchanpur | Sudur Pashchim |
|  | 6 | Bedkot NP | Kanchanpur | Sudur Pashchim |
|  | 12 | Suklaphanta NP | Kanchanpur | Sudur Pashchim |
|  | 6 | Krishnapur NP | Kanchanpur | Sudur Pashchim |
|  | 9 | Punarbas NP | Kanchanpur | Sudur Pashchim |
|  | 6 | Godawari NP | Kailali | Sudur Pashchim |
|  | 1 | Dhangadi Upamaha | Kailali | Sudur Pashchim |
|  | 12 | Dhangadi Upamaha | Kailali | Sudur Pashchim |
|  | 1 | Gauriganga NP | Kailali | Sudur Pashchim |
|  | 7 | Gauriganga NP | Kailali | Sudur Pashchim |
|  | 4 | Chure GP | Kailali | Sudur Pashchim |
|  | 3 | Ghodaghodi NP | Kailali | Sudur Pashchim |
|  | 1 | Bardagoriya GP | Kailali | Sudur Pashchim |
|  | 1 | Tikapur NP | Kailali | Sudur Pashchim |
|  | 2 | Tikapur NP | Kailali | Sudur Pashchim |
|  | 3 | Janaki GP | Kailali | Sudur Pashchim |
|  | 10 | Birendranagar NP | Surkhet | Karnali |
|  | 11 | Birendranagar NP | Surkhet | Karnali |
