# Supplementary material for: Analyzing Spatial and Temporal Patterns of Designated Malaria Risk Areas in Nepal from 2018 to 2021
Source: Vector Borne Zoonotic Dis. 2023 Jun 5;23(6):350–3. doi: 10.1089/vbz.2022.0097 (PMC10278016; doi:10.1089/vbz.2022.0097)
Supplement: Supplemental data [file Supp_TableS2.docx]

**Table S2: Number of High-Risk (HR) wards with STAMP Analysis**

|  | 2018-2019 | 2019-2020 | 2020-2021 |
| --- | --- | --- | --- |
| Disappeared HR | 14 | 26 | 11 |
| Generated HR | 12 | 7 | 5 |
| Stable HR | 35 | 21 | 17 |
