# Supplementary material for: Analyzing Spatial and Temporal Patterns of Designated Malaria Risk Areas in Nepal from 2018 to 2021
Source: Vector Borne Zoonotic Dis. 2023 Jun 5;23(6):350–3. doi: 10.1089/vbz.2022.0097 (PMC10278016; doi:10.1089/vbz.2022.0097)
Supplement: Supplemental data [file Supp_TableS3.docx]

**Table S3: Number of Moderate-Risk (MR) wards with STAMP analysis**

|  | 2018-2019 | 2019-2020 | 2020-2021 |
| --- | --- | --- | --- |
| Disappeared MR | 52 | 53 | 72 |
| Generated MR | 50 | 26 | 16 |
| Stable MR | 101 | 98 | 52 |
